# Differential expression of cyclins CCNB1 and CCNG1 is involved in the chondrocyte damage of kashin-beck disease

**DOI:** 10.3389/fgene.2022.1053685

**Published:** 2022-12-14

**Authors:** Kaidiriye Setiwalidi, Jialei Fu, He Hei, Shaniya Nuer, Feiyu Zhang, Sijie Chen, Yanli Liu, Feihong Chen, Shujin Li, Chaowei Wang, Yifan Wu, Yi Gong, Minhan Hu, Ruitian Huang, Junyi Liu, Tianxiao Zhang, Yujie Ning, Hongmou Zhao, Xiong Guo, Xi Wang

**Affiliations:** ^1^ School of Public Health, Xi’an Jiaotong University Health Science Center, Key Laboratory of Trace Elements and Endemic Diseases, Collaborative Innovation Center of Endemic Disease and Health Promotion for Silk Road Region, Xi’an, China; ^2^ Department of Occupational and Environmental Health, School of Public Health, Xi’an Jiaotong University Health Science Center, Xi’an, China; ^3^ Department of Epidemiology and Health Statistics, School of Public Health, Xi’an Jiaotong University Health Science Center, Xi’an, China; ^4^ Foot and Ankle Surgery Department, Honghui Hospital of Xi’an Jiaotong University, Xi’an, China; ^5^ Clinical Research Center for Endemic Disease of Shaanxi Province, The Second Affiliated Hospital of Xi’an Jiaotong University, Xi’an, China

**Keywords:** kashin-beck disease (KBD), chondrocyte, cyclin, CCNB1, CCNG1

## Abstract

The purpose of this study was clarify the relationship between the differential expression of cyclins CCNB1 and CCNG1 and chondrocyte damage in Kashin-Beck disease. Systematic review and high-throughput sequencing of chondrocytes derived from Kashin-Beck disease patients were combined to identify the differentially expressed cyclins and cyclin-dependent kinase genes. In parallel, weaned SD rats were treated with low selenium for 4 weeks and then T-2 toxin for 4 weeks. Knee cartilage was collected to harvest chondrocytes for gene expression profiling. Finally, the protein expression levels of CCNB1 and CCNG1 were verified in knee cartilage tissue of Kashin-Beck disease patients and normal controls by immunohistochemical staining. The systematic review found 52 cartilage disease-related cyclins and cyclin-dependent kinase genes, 23 of which were coexpressed in Kashin-Beck disease, including 15 upregulated and 8 downregulated genes. Under the intervention of a low selenium diet and T-2 toxin exposure, CCNB1 (FC = 0.36) and CCNG1 (FC = 0.73) showed a downward expression trend in rat articular cartilage. Furthermore, compared to normal controls, CCNB1 protein in Kashin-Beck disease articular cartilage was 71.98% and 66.27% downregulated in the superficial and middle zones, respectively, and 12.06% upregulated in the deep zone. CCNG1 protein was 45.66% downregulated in the superficial zone and 12.19% and 9.13% upregulated in the middle and deep zones, respectively. The differential expression of cyclins CCNB1 and CCNG1 may be related to articular cartilage damage in Kashin-Beck disease.

## Introduction

Kashin-Beck disease (KBD) is an endemic deformed osteoarthropathy with unknown etiology and pathogenesis that is mainly distributed in the low-selenium region from northeast to southwest China, Siberia, North Korea, and Russia (Luo et al., 2011). According to the 2020 China Health Statistics Yearbook, there are 379 endemic districts and counties with over 103 million residents at risk (www.nhc.gov.cn). The disease mainly affects children and adolescents during skeletal development. Clinical manifestations include arthralgia, morning stiffness, joint deformation, limited movement, short limbs and even short stature (Li et al., 2018). This not only directly affects the patients’ quality of life and restricts their family economic development but is also one of the main public health problems in which illness causes poverty (Yamamuro, 2001).

KBD has been identified as a gene-environment disease. Environmental selenium deficiency and mycotoxin contamination in food are the main risk factors for the disease (Chasseur et al., 1997; Sun et al., 2010; Fairweather-Tait et al., 2011). However, the pathogenic mechanism of these exposures is unknown. The primary lesions are necrosis and excessive apoptosis of chondrocytes in epiphyseal plate cartilage and articular cartilage (Wang et al., 2019). The apoptosis and necrosis of cells are regulated by the cell-cycle center mechanism, and several studies have shown that cyclins and cyclin-dependent kinases (CDKs) play an important role in the coordination of chondrocyte proliferation and differentiation (Beier et al., 1999). Previous studies have proven that the dysregulation of cyclins and CDKs is involved in the development of osteoarthritis (OA). For example, CDK9 is highly expressed in vitro and *in vivo* inflammatory models, and its specific inhibitor LDC067 prevents cartilage destruction by inhibiting IL-1β induced NF-κB signaling pathway activation in chondrocytes (Xue et al., 2019). CDKN1A induces MAPK8 to participate in the inactivation of MAPK by activating the AKT pathway, further reducing chondrocyte apoptosis (Xie et al., 2021), and knockdown of the lncRNA RMRP promotes proliferation and inhibits apoptosis of OA chondrocytes through the miR-206/CDK9 axis (Lu et al., 2020). A number of studies have confirmed that the NF-κB signaling pathway and AKT pathway induced by IL-1β are also involved in the pathogenesis of KBD (Cao et al., 2008; Yan et al., 2010; Tang et al., 2012; Huimin Wang et al., 2014; Zhou et al., 2014), indicating that there are similarities between KBD and the pathological process of osteochondropathies regulated by cyclins and CDKs. Therefore, dysregulation of cyclins and CDKs may be associated with excessive apoptosis and necrosis of KBD chondrocytes.

In recent years, studies on the pathogenesis of KBD have found a number of abnormally expressed genes related to KBD, whose functions are mostly involved in metabolism, signal transduction, membrane transport (ion channel transport proteins) and immunoregulation (Wang et al., 2013). However, few studies have focused on the relationship between cyclins and chondrocyte damage in KBD.

Therefore, this study integrated a systematic review and high-throughput sequencing analysis of chondrocytes to identify cyclins and CDKs related to chondrocyte damage in KBD. Then, SD rats treated with a lowselenium diet followed by T-2 toxin were sacrificed to collect knee cartilage for high-throughput sequencing to verify the expression of candidate genes. Finally, the protein expression levels of candidate genes in KBD patients and normal controls were verified by immunohistochemistry to clarify the relationship between candidate cyclins and cartilage damage in KBD. The results provide new evidence for the role of environmental exposures in the pathogenesis of KBD.

## Materials and methods

### Systematic review

PubMed, Wanfang and CNKI databases were mainly used to perform literature research. Keywords including osteochondropathy, chondrocytes, and cyclins in both English and Chinese were put in to collect cyclins and CDKs that differentially expressed in various osteochondropathies.

### Subjects and sample collection

The diagnosis and classification of patients with KBD were carried out in strict accordance with the national standard [WS/T207-2010], briefly based on the clinical manifestations and the frontal X-ray image of right hand. Subjects were excluded if they 1) have been living in the endemic areas less than 5 years; 2) were suffering/had suffered from any other osteoarthropathy including osteoarthritis, rheumatoid arthritis, gout, skeletal fluorosis any other type of osteochondral dysplasia, and any chronic diseases, which cause necessity to receive any treatment that may affect the bone within the past 6 months.

The knee cartilage tissue was obtained for cell culture and immunohistochemistry. All articular cartilage samples, including all of the cartilage zones (including calcified) and subchondral bone, were harvested from the lateral tibial plateau and obtained within 1 h after operation. The subjects included four advanced KBD patients (42–55 years old) and four normal controls (37–55 years old), with a male to female ratio of 3:1. All of KBD patients underwent arthroplasty due to severe cartilage damage and normal subjects due to traffic or other accidents. This study was approved by the ethics committee of Xi’an Jiaotong University (no. 2018-206). Cartilage samples were collected after obtaining the informed consent of patients and their families. The male SD rats used in this experiment were from the animal experiment center of the medical department of Xi’an Jiaotong University.

### Primary cell culture

Tissue samples, including cartilage areas and 0.5–1.0 cm subchondral bone were taken from the lateral tibial plateau and collected within 1 h after operation. Chondrocytes were isolated as previous publications (Tong et al., 2019; Yao et al., 2021). Briefly, articular cartilage samples were washed twice with sterile phosphate buffered saline (PBS) and antibiotics (0.1% penicillin and streptomycin). Then cut the tissue into 1 mm^3^ small pieces, and digested with 0.25% trypsin enzyme at 37°C and 5% CO_2_ for 30 min. Cell suspension was centrifuged at 1000 × g for 5 min, collected the supernatant and maintained the temperature at 37°C with Eppendorf thermal mixer, and digested the cells in the medium supplemented with 0.2% type II collagenase for 12–16 h. The isolated chondrocytes were filtered through 70 mm nylon filter, washed twice with sterile PBS, and then inoculated into T_25_ cell culture flask, 5%CO_2_, 37°C incubator. Observe the growth of chondrocytes every day, and change the culture medium every 3 days.

### Total RNA extraction

Digest and collect chondrocytes, place them into centrifuge tubes, and add 10 cm^2^/ml pyrolysis solution to react at room temperature for 5 min. Next, add 4°C trichloromethane, followed by vigorous oscillation, and leave it at room temperature for 3 min. Then, centrifuge by 12000 rpm at 4°C for 15 min, and abstract the supernatant. Add 4°C isopropyl alcohol to react at room temperature for 30 min, followed by 4°C, 12000 rpm/min, centrifuging for 10 min. Discard the supernatant and wash the sediment with 75% alcohol at 4°C. After washing, centrifuge with speed of 8000 rpm at 4°C for 10 min, discard the supernatant, vacuum dry the RNA precipitation. Then, add RNase-free water, gently blow and rinse the precipitation, and place it in a 50°C water bath for 10 min. After completely dissolving the RNA precipitation, part of the RNA is used for high-throughput sequencing according to manufacture of Agilent microarray analysis (Li et al., 2012), and the remaining RNA is stored at −80°C for later experiment.

### *In vivo* experiment of selenium-deficiency and T-2 toxin combined toxication

Randomly divided 12 male weaned rats (3 w, 30–50 g) into control group and low selenium group, according to the previous study (He et al., 2016), we separately fed with control diet (selenium content of 0.18 ppm) and low selenium diet (selenium content of 0.02 ppm, Nantong Luofei feed company, China). Four weeks later, three rats in each group were randomly selected and collected blood to determine the selenium status in rats (Supplementary Figure S1). Next, the low selenium group continued to be given a dose of 200 ng/g × BW/d T-2 toxin (cas:21259-20–1, Beijing bailingwei Technology Co., Ltd, China), and the control group was administered by gavage with 0.9% normal saline of the same dose conversion volume (Liu et al., 2021). Four weeks later, 0.5ml/100g × BW dose of 10% chloral hydrate was injected intraperitoneally to anesthetize rats 24 h after the last gavage. The blood was collected from abdominal aorta and the rats were sacrificed afterwards to collect knee cartilage for detection of candidate genes.

### Hematoxylin-eosin (HE) staining

To verify the combined toxic effect of low-selenium and T-2 toxin, femoral articular cartilage of rats was collected for HE staining as described in our previous publication (Wang et al., 2017). First, articular cartilage samples were fixed in 4% (w/v) paraformaldehyde at 4°C. Next, decalcified the fixed cartilage in 10% (w/v) EDTA disodium salt for 4 weeks with regular agitation. After paraffin embedding, the cartilage was cut into serial sections, stained with H&E, and examined microscopically.

### Immunohistochemical staining verification

Cartilage tissues were fixed with 4% (w/v) paraformaldehyde for 24 h immediately after acquisition and decalcified in 10% (w/v) ethylene diamine tetra-acetic acid disodium salt (EDTA-Na2) for 2–3 weeks. The samples were dehydrated in a series of alcohol, cleared in xylene, and embedded in paraffin wax. Paraffin sections were cut into 5-µm sections, mounted on slides, and stored at room temperature until used for stainings. Prepare 5 μm-thick cartilage sections for immunohistochemistry staining according to the following processes. The sections were firstly dewaxed and hydrated by being orderly immersed in anhydrous ethanol for 10min, 95% ethanol for 5 min, 80% ethanol for 5 min, 70% ethanol for 5 min. Next, the sections were washed with distilled water for 5 min, and incubated with hydrogen peroxide solution for 5–10 min to inactive endogenous peroxidase. After this, the sections were incubated with urea (10 mol/L) and compound digestive solution in 37°C incubator for 20 min for antigen retrieval. In the following, goat serum was blocked at 37°C for 20 min, and then incubated with CCNG1 and CCNB1 primary antibodies (Rabbit anti human, a5292, a16800, pierce biotechnology, United States) at 4°C overnight. Then, incubated the sections with horseradish peroxidase labeled CCNG1 and CCNB1 second antibodies (Beijing zhongshanjinqiao rabbit SP kit, sp-9001) at 37°C for 20 min, and horseradish peroxidase labeled streptavidin working solution for 15 min. Finally, the sections were stained with DAB kit. Pictures were taken by Leica film system. Assessment of the staining throughout each cartilage zone included systematic counting of positive and negative cells, starting from the cartilage surface and progressing down through all layers of cartilage. The percentage of positive cells was calculated using the number of positively stained cells divided by the total number of cells (positively and negatively stained cells) in the chosen fields of view. The percentages of positive cells in different zones were calculated for each case and then for the different groups.

### Statistical analyses

SPSS17.0 is used for statistical analysis. The results were expressed as mean ± standard deviation (‾x ± SD). The data are first tested for normal distribution and homogeneity of variance. If the data are normally distributed with homogeneous variance, *t*-test is used for the comparison of two groups of data, one-way ANOVA for the comparison among multiple groups, and LSD-*t* test for multiple comparisons. Otherwise, the non-parametric rank sum test is used for comparisons. All statistical tests are bilateral tests, and *p* < 0.10 indicates that the difference is statistically significant.

## Results

### Differential expression and function of cyclins and CDKs in osteochondropathies

A systematic review found that 52 cyclins and CDKs were involved in the occurrence and development of various diseases. Among them, six cyclins and CDKs were associated with osteochondropathies, including CCNB1, CCNG1, CDK5, CDKN1C, CDK19, and CDK10, as detailed in Table 1.

**TABLE 1:** Role of cyclins and CDKs in the development of osteochondropathies.

Gene	Full name	Gene function (PMID)
CCNB1	cyclin B1	• CCNB1-PKD1 gene pair is a molecular marker for identifying oxidative stress in intervertebral disc degeneration (IDD) (34858418)
		• CCNB1 is involved in the pathogenesis of osteosarcoma (35032258)
		• Low expression of CCNB1 inhibits the proliferation of fibroblasts in patients with hip dysplasia (DDH) (34465146)
CCNG1	cyclin G1	• The inhibitory effect of FXR on osteosarcoma (OS) cell proliferation and growth is highly correlated with down-regulating CCNG1 levels (31779647)
		• Ectopic miR-122 downregulates the expression of CCNG1 in U2OS osteosarcoma cells, inhibits cell proliferation and migration to induce apoptosis (25269820)
CDK5	cyclin-dependent kinase 5	• CDK5 promotes fracture healing through the Erk1/2 pathway (35383146)
		• Inhibition of CDK5 reduces bone loss (35203613)
		• CDK5 is overexpressed in osteosarcoma (28323497)
		• Increase in the protein and activity of CDK2/CDK5 is involved in osteosarcoma cells (23042366)
CDKN1C	cyclin-dependent kinase inhibitor 1C	• Mutation IDH1-mediated SOX9 and CDKN1C expression regulates chondrosarcoma tumor progression (31406254)
		• Loss of function variant in CDKN1C is associated with Beckwith-Wiedemann syndrome (31497289)
		• CDKN1C mutations are associated with growth disorders (25057881)
CDK19	cyclin-dependent kinase 19	• Inhibition of CDK8/19 in osteoblasts promotes osteoblast mineralization (30793301)
CDK10	cyclin-dependent kinase 10	• CDK10 regulates cell growth (28886341)
		• CDK10 mutation causes growth retardation, spinal deformities, facial deformities, and intellectual disability (34369103)

### Expression of cyclins and CDKs in KBD chondrocytes

The results of gene expression profiling showed that 23 of the 52 cyclins and CDKs found in the systematic review were commonly expressed in KBD chondrocytes, of which CCNB1, CCNG1, CCNG2, CDK5RAP2, CDNK1, and CDKN3 had difference in expression >1.5 times that of normal chondrocytes, as shown in Table 2.

**TABLE 2:** Expression of cyclins and CDKs in KBD chondrocytes.

Gene	Differential expression (FC)	
	Mean	Scope
CCNB1	1.62	0.50–4.21
CCNB2	0.80	0.40–1.44
CCND1	1.40	0.55–2.81
CCND2	0.91	0.43–2.17
CCND3	0.78	0.23–1.51
CCNE1	1.22	1.04–1.48
CCNG1	2.73	0.39–7.07
CCNG2	1.84	0.40–3.57
CCNH	0.91	0.56–1.89
CCNI	1.47	0.51–3.44
CDK10	0.79	0.10–1.68
CDK2	0.80	0.31–1.36
CDK4	1.33	0.61–2.99
CDK5	1.05	0.38–1.64
CDK5RAP2	1.84	0.56–2.34
CDK7	1.48	0.52–2.53
CDKL1	1.05	0.43–2.45
CDKL3	0.92	0.37–2.35
CDKN1 A	1.43	0.58–1.86
CDKN1 C	2.22	0.71–5.08
CDKN2A	0.74	0.31–1.13
CDKN2C	1.04	0.61–1.49
CDKN3	1.75	0.56–3.17

### HE staining of articular cartilage derived from rats fed a low selenium diet and exposed to T-2 toxin

**FIGURE 1:**
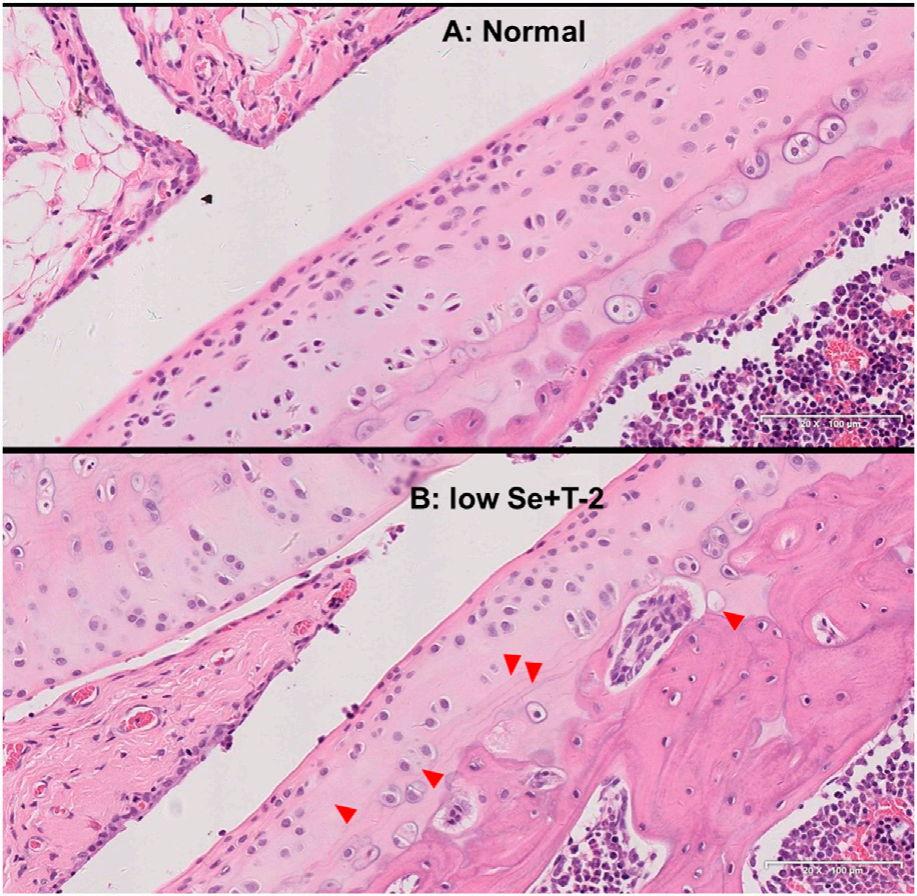
Morphology of articular cartilage of rats. Note: **(A)** normal diet group; **(B)** low selenium and T-2 toxin group. Red triangle points abnormal pathological change.

In the normal diet group, the rat cartilage exhibited a clear hierarchical structure with healthy morphology (Figure 1A). In the low selenium diet and T-2 toxin exposure groups, the rat cartilage showed typical pathological morphology, including a dramatic decrease in chondrocytes, multiple calcification lines, nuclei showing extreme pyknosis or disappearance in the deeper zone, and necrosis presented by vacuoles (Figure 1B).

### Expression profile of knee chondrocytes obtained from rats fed a low-selenium diet and poisoned with T-2 toxin

The results of gene expression profiling of rat articular chondrocytes showed a downward expression trend. CCNB1 (FC = 0.36, *p* = 0.12), CCNG1 (FC = 0.73, *p* = 0.21), CDK5 (FC = 0.86, *p* = 0.57), and CDKN1C (FC = 0.78, *p* = 0.21) in the risk factor-exposed group compared with the control group, as detailed in Table 3.

**TABLE 3:** Expression of candidate genes in chondrocytes derived from low selenium and T-2 toxin treated rats.

Genes	‾x±SD		Differential expression (FC)	*p*-value
	Control group	T-2 toxin and low selenium group		
CCNB1	3.92 ± 8.20	1.40 ± 2.07	0.36	0.12
CCNG1	445.47 ± 102.42	325.50 ± 89.80	0.73	0.21
CDK5	6.23 ± 1.05	5.38 ± 0.57	0.86	0.57
CDKN1C	57.83 ± 14.21	45.27 ± 22.49	0.78	0.21

‾x±SD, devotes mean ± standard deviation; Differential expression was the expression of target genes in the experimental group compared with the control group.

### Expression of CCNB1 and CCNG1 proteins in articular cartilage in patients with KBD

**FIGURE 2:**
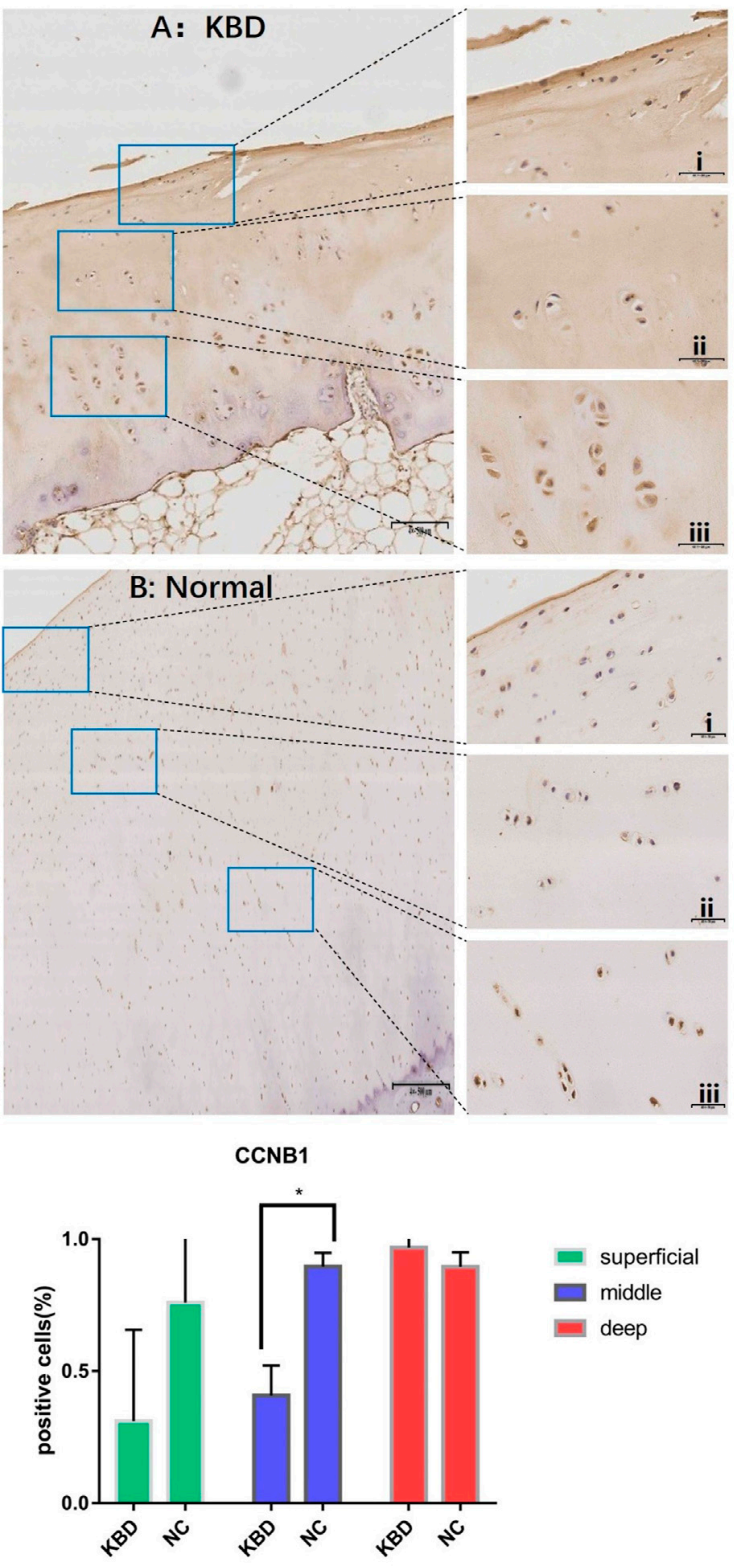
CCNB1 IHC results and positive staining percentage in the superficial/middle/deep zone of KBD patients and normal human cartilage tissues. Notes: **(A)** Chondrocytes from patients with KBD; **(B)** Normal human chondrocytes; i represents the superficial zone, ii represents the middle zone, and iii represents the deep zone; *Indicates *p* < 0.10, means the difference between the two is statistically significant.

**FIGURE 3:**
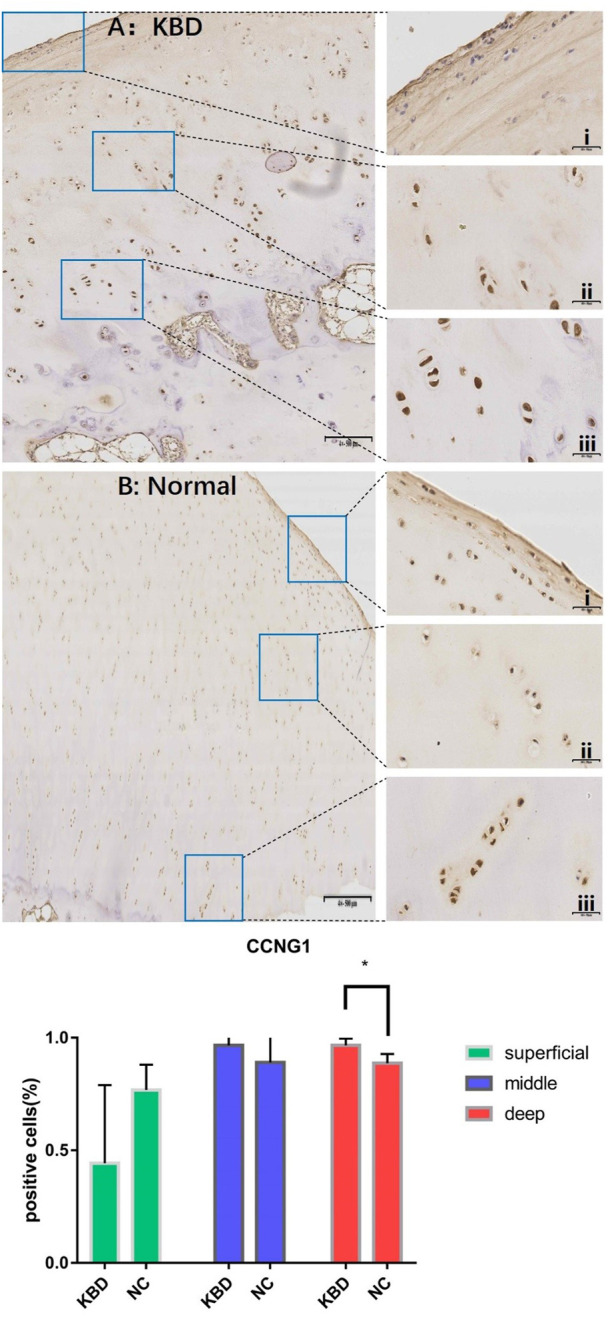
CCNG1 IHC results and positive staining percentage in the superficial/middle/deep zone of KBD patients and normal human cartilage tissues. Notes: **(A)** Chondrocytes from patients with KBD; **(B)** Normal human chondrocytes; i represents the superficial zone, ii represents the middle zone, and iii represents the deep zone; *Indicates *p* < 0.10, means the difference between the two is statistically significant.

In combination with cyclin expression and function in bone and chondrocytes, high-throughput sequencing and animal experiment results, CCNB1 and CCNG1 were selected as validated genes. Immunohistochemical staining of KBD articular cartilage showed that compared with normal controls, CCNB1 protein was 71.98% and 66.27% downregulated in the superficial (F = 2.265, *p* = 0.229), and middle zones (F = 47.328, *p* = 0.006), respectively, and 12.06% upregulated in the deep zone (F = 2.596, *p* = 0.205), as detailed in Figure 2. CCNG1 protein was 45.66% downregulated in the superficial zone (F = 2.395, *p* = 0.197)and 12.19% and 9.13% upregulated in the middle (F = 1.136, *p* = 0.347) and deep zones (F = 7.368, *p* = 0.053), respectively, as shown in Figure 3.

## Discussion

To date, the mechanism of low selenium status and T-2 toxin exposure in the pathogenesis of KBD not been determined. This study integrated a systematic review, gene expression profiling, animal experiments and target tissue immunohistochemical verification to show that a low selenium diet and T-2 toxin exposure may induce cell cycle arrest by regulating the expression of cyclins, such as CCNB1, CCNG1, CDK5, and CDKN1C, and CDKs through regulation of the apoptosis mechanism of chondrocytes. These changes may be related to regulators of apoptosis, such as BCL-2 and Fas p53, as well as epigenetic variation.

Cyclin B1 (CCNB1) appears in S phase, mainly in the cytoplasm, enters the nucleus at the onset of mitosis and is actively transcribed during the mitotic stage (Pines and Hunter, 1992; Sciortino et al., 2001), which is an important part of the cell-cycle pathway. Regulating apoptosis is one of the most important functions of CCNB1. Silencing CCNB1 can inhibit cell proliferation and promote cell senescence by activating the p53 signaling pathway (Zhang et al., 2018). Other evidence showed that downregulation of the CCNB1 gene resulted in a significant decrease in cell viability and proliferation, cell cycle arrest in the G2/M phase, and increased apoptosis (Li et al., 2019). In this study, CCNB1 was downregulated in the superficial and middle zones of KBD cartilage, inhibiting cell proliferation and causing cell stagnation, which is consistent with the above experimental results, while upregulation in the deep zone could explain the accelerated necrosis of focal chondrocytes. The mechanism may that under the influence of selenium induced oxidative stress, the changes of Cdc25c and p21 mediate the downregulation of CDC2/CCNB1 complex (Kaushal and Bansal, 2007), and toxin exposure regulates the expression of CCNB1 protein (Chang et al., 2016), which trigger cell cycle arrest, hinder or delay the synthesis of cell DNA, and accelerate cell apoptosis.

Cyclin G1 (CCNG1), as a member of the g-type cyclins, is located on chromosome 5q-32-q34 and consists of 259 amino acids (Gordon and Hall, 2009); it is also one of the earliest discovered p53 target genes (Okamoto et al., 2002). P53 was found to be upregulated by selenium deficiency and T-2 toxin exposure, which is associated with the severity of articular cartilage damage in KBD patients and experimental rats (Yang et al., 2017; Yang et al., 2022). Studies have confirmed that CCNG1 is overexpressed in osteosarcoma tissues (Han et al., 2018), and the inhibition or knockout of CCNG1 can inhibit cell proliferation and colony formation, indicating that CCNG1 negatively regulates cell growth (Chen et al., 2020). A study by OKAMOTO K et al. found that CCNG1 cannot induce the cell death program *de novo* under normal conditions but made cells prone to apoptosis when induced by other signals such as serum starvation (Okamoto and Prives, 1999). The significantly upregulated expression of CCNG1 in the deep zone of articular cartilage in KBD patients suggests that CCNG1 may accelerate the chondrocyte necrosis of deep cartilage induced by damage factors. Our previous research found that low selenium status and T-2 toxin exposure can affect the gene and protein expression levels of PPARG, and its SNP rs12629751 is associated with KBD (Ning et al., 2021). PPARG ligands influence the activities of CCNG1, CDK1, GADD45β, CCNA1 and ATM proteins, which are involved in the LPS-triggered expression of genes controlling the DNA damage response (Mierzejewski et al., 2022). Under mycotoxin treatment, correlations are found between two representative miRNAs (hsa-miR-1-3p and hsa-miR-122-5p) and their target genes PDCD10 and CCNG1, which are associated with apoptotic signaling and the cell cycle (Gong et al., 2019). T-2 toxin is a common mycotoxin and has been identified as a significant environmental risk factor for KBD. This evidence combined with T-2 toxin resulting in decreased expression of CCNG1 mRNA (Lu et al., 2021) suggests that selenium deficiency and T-2 toxin exposure could cause and accelerate chondrocyte apoptosis and necrosis in KBD cartilage partly through regulating CCNB1 and CCNG1.

In addition, although there was a lack of immunohistochemical verification, CDKs, such as CDK5 and CDKN1C, also showed gene expression differences in KBD compared with normal controls in all cell and animal sequencing analyses. Cyclin-dependent kinase 5 (CDK5) is a member of a family of serine/threonine kinases involved in regulating the progression of different stages of the eukaryotic cell cycle (Morgan, 1997) and plays a role in cell proliferation, apoptosis, inflammation and the immune response (Do and Lee, 2020). CDK5 can be activated by cyclin I and achieve anti-apoptotic function through the increased expression of BCL-2 family proteins (Gupta and Singh, 2019). Selenium deficiency and T-2 toxin exposure downregulate the BCL2 gene and protein in KBD chondrocytes and cartilage tissue. Decreased BCL2 leads to increased chondrocyte apoptosis (Yang et al., 2022). In other words, downregulation of CDK5 expression could induce chondrocyte apoptosis. The latest evidence also supports the involvement of CDK5 in transcriptional, post transcriptional and post translational modifications to regulate cell cycle progression (Cortés et al., 2019); thus, it is reasonable to hypothesize that it plays similar roles in chondrocytes. Dietary selenium can significantly alter the expression of a group of genes, including CCND1, CDK5, CDK4, CDK2, CDC25 A and GADD153, to regulate cell apoptosis (Narayanan, 2006). In addition to selenium deficiency, T-2 toxin can also increase the mRNA expression of CDK5 (Lu et al., 2021).

Cyclin-dependent kinase inhibitor 1C (CDKN1C) can tightly bind and inhibit the cyclin/CDKs complex and then regulate the progression of the cell division cycle (Stampone et al., 2018). CDKN1C is a key negative regulator of growth (Suntharalingham et al., 2019) and plays an important role in epigenetic studies. Excessive apoptosis of chondrocytes may be related to CDKN1C downregulation in early chondrocyte dysplasia and epigenetics. Selenium results in decreased mRNA expression of CDKN1C (Tsavachidou et al., 2009). Methylselenol, a selenium metabolite, inhibited cell growth by upregulating the mRNA levels of CDKN1C, heme oxygenase 1, platelet/endothelial cell adhesion molecule, and PPARG genes, which are directly related to the regulation of the cell cycle and apoptosis. The regulation of these genes is likely to play a key role in G1 cell cycle arrest and apoptosis (Zeng et al., 2009). These controversial findings indicate that selenium could play a double-edged role in maintaining the cell cycle and apoptosis by working with cyclins and CDKs. There must be an optimum balance between selenium and cyclins/CDKs, and it needs to be clarified.

This study explored the role of cyclin and CDK in the cartilage damage of KBD induced by a low selenium diet and T-2 toxin exposure through a systematic review and high-throughput sequencing, cell and risk-factor treated animal experiments, and target tissue verification. However, due to the difficulty in obtaining articular cartilage samples from KBD patients and healthy controls, the sample size of the immunohistochemistry study was relatively small. Therefore, to verify that the expression difference of candidate proteins has an upward or downward trend without significant statistical significance, we reduced the experimental error using the double criteria of accurate count of positive cells and degree of staining and repeated the calculation of percentage of positive cells. In addition, little research has focused on the role of cyclins and CDKs in osteoarthropathies, and their alterations in KBD chondrocytes under the stimulation of a low selenium diet and T-2 toxin exposure may be different from other diseases. Therefore, these genes may play a double-edged role in the regulation of cell proliferation and apoptosis, which needs to be further studied.

## Conclusion

Low selenium status and T-2 toxin exposure can interfere with the expression of cyclins and CDKs. The damage to articular cartilage in KBD may be related to the differential expression of CCNB1 and CCNG1, which provides new evidence for the pathogenesis of KBD.

## Data Availability

The raw data supporting the conclusions of this article will be made available by the authors, without undue reservation.

## Abbreviations

KBD, Kashin-Beck Disease; CDKs, cyclin-dependent kinases; OA, osteoarthritis; CCNB1, Cyclin B1; CCNG1, Cyclin G1; PBS, phosphate buffered saline; HE, Hematoxylin-eosin.
