# Supplementary material for: Differential expression of cyclins CCNB1 and CCNG1 is involved in the chondrocyte damage of kashin-beck disease
Source: Front Genet. 2022 Dec 14;13:1053685. doi: 10.3389/fgene.2022.1053685 (PMC9794764; doi:10.3389/fgene.2022.1053685)
Supplement: Supplementary file 1 [file Table1.DOCX]

**Supplementary Figure legends：**

**Supplementary Figure 1** Selenium concentration in serum of rats. * means P<0.05
